# Cigarette smoke exposure triggers dendritic cell-derived exosome-mediated Th17 and Treg polarization through an autophagy- and necroptosis-associated SIRT1-dependent mechanism in vitro

**DOI:** 10.3389/fimmu.2025.1715736

**Published:** 2026-01-06

**Authors:** Dongling Luo, Jifeng Liu, Leilei Ya, Chengxuan Ruan, Siyi Ou, Yiqian Wei, Qingyun Lin, Zhaohong Huang, Kaiwen Nong

**Affiliations:** 1 Department of Pulmonary and Critical Care Medicine, The First People’s Hospital of Qinzhou/Tenth Affiliated Hospital of Guangxi Medical University, Qinzhou, China; 2 Department of Pulmonary and Critical Care Medicine, Liuzhou Municipal Liutie Central Hospital, Liuzhou, China; 3 Department of Pulmonary and Critical Care Medicine, The Ninth Affiliated Hospital of Guangxi Medical University, Beihai, China

**Keywords:** exosomes, dendritic cell, cigarette smoke extract, silent information regulator 2 homolog 1, CD4+ T cells

## Abstract

**Introduction:**

Both Th17/Treg cell imbalance and dendritic cells (DCs) play critical roles in chronic obstructive pulmonary disease pathogenesis from cigarette smoke. Previous studies have shown that DC-derived exosomes (DCexos) can polarize CD4^+^ T cells toward either Th17 or Treg phenotypes. However, the role of SIRT1 in regulating DCexos-mediated immune responses under cigarette smoke extract (CSE) exposure, and its association with autophagy and necroptosis, remains unclear. In this study, we assessed the expression of silent information regulator 2 homolog 1 (SIRT1), autophagy markers (ATG16L1, LC3B, and p62/SQSTM1), and necroptosis markers (ZBP1, RIPK3, MLKL, Caspase-8, and Caspase-3) in DCs following CSE exposure. Additionally, we evaluated the effect of a SIRT1 activator (SRT1720) on CSE-exposed DCexos and its ability to polarize CD4^+^ T cells toward Th17 and Treg subsets.

**Methods:**

DCs were generated from bone marrow-derived mononuclear cells isolated from C57BL/6J mice and assigned to three groups: control DCs, CSE-exposed DCs, and SRT1720-treated CSE-exposed DCs. The ability of each group’s exosomes to polarize CD4^+^ T cells was assessed using a mixed lymphocyte reaction (MLR).

**Results:**

SIRT1 expression in CSE-exposed DCs decreased in a time-dependent manner (all *p* < 0.05). Expression of ATG16L1 and p62 was also reduced, while LC3B expression was increased under CSE-exposed (all *p* < 0.01). Expression of ZBP1, RIPK3, MLKL, Caspase-8, and Caspase-3 was elevated following CSE exposure (all *p* < 0.01). Th17 cell frequencies were increased in the CSE-exposed DC-derived exosomes/MLR group compared to the controls (*p* < 0.01), while Treg frequencies were decreased (*p* < 0.01). Autophagy could be improved and the necroptosis could be reduced in the SRT1720-treated CSE-exposed DCs (all *p* < 0.01). Additionally, Th17 cell polarization reduced and Treg cell differentiation increased in the SRT1720-treated CSE-exposed DC-derived exosomes/MLR group compared to the CSE-exposed group (all *p* < 0.01).

**Conclusion:**

CSE exposure induces an imbalance in Th17/Treg polarization through a process mediated by DCexos that entails reduced SIRT1 expression, increased necroptosis, and dysregulated autophagy. SIRT1 activation by SRT1720 can attenuate these effects by restoring immune balance and modulating cell death and survival pathways in DCs under CSE exposure.

## Introduction

Chronic obstructive pulmonary disease (COPD) is a chronic respiratory disorder that is prevalent throughout the globe characterized by high morbidity and mortality, posing significant public health and socioeconomic challenges. The number of individuals diagnosed with COPD is expected to reach approximately 600 million by 2050 (1). Despite its widespread impact, progress in understanding the mechanisms driving COPD progression and mortality has been limited (2–4).

Cigarette smoking remains the most prominent environmental risk factor for COPD, inducing chronic, nonspecific inflammation that plays a pivotal role in both disease initiation and progression (5, 6). Recent immunological studies have highlighted the involvement of T-cell-mediated inflammation, particularly the imbalance between pro-inflammatory Th17 cells and anti-inflammatory regulatory T (Treg) cells, as a key element in COPD pathogenesis (7–9). Dendritic cells (DCs), as central regulators of adaptive immunity, contribute to this imbalance by presenting antigens and secreting cytokines that influence CD4^+^ T cell differentiation (7, 8, 10–12). Emerging research indicates that DC-derived exosomes significantly influence CD4^+^ T cell polarization toward Th17 or Treg phenotypes. Exosomes are small extracellular vesicles containing various bioactive molecules, including proteins, lipids, and nucleic acids, capable of modulating recipient cell behavior upon uptake (13–15). In the context of cigarette smoke exposure, DC-derived exosomes have been shown to skew CD4^+^ T cell differentiation toward pro-inflammatory Th17 and Th1 subsets, thereby exacerbating inflammation in COPD (16, 17).

The biogenesis and function of exosomes are regulated by multiple molecular mechanisms. Silent information regulator 2 homolog 1 (SIRT1), an NAD^+^-dependent deacetylase, has been implicated in the regulation of exosome formation and secretion (18). Furthermore, Z-DNA binding protein 1 (ZBP1) has been identified as a key regulator of necroptosis, autophagy, and exosome release, playing a central role in cellular homeostasis. Necroptosis is a programmed form of necrotic cell death distinct from apoptosis that is mediated by ZBP1 through its interactions with receptor-interacting protein kinase 3 (RIPK3) and mixed lineage kinase domain-like pseudokinase (MLKL). This pathway is particularly activated by viral infection and inflammatory signals (19–21). Conversely, autophagy, typically considered a cytoprotective process, has been shown to either promote or inhibit necroptosis depending on the cellular context. ZBP1 has also been implicated in regulating autophagy-related pathways, further highlighting its critical role in determining cell fate under stress conditions (22–24). In COPD, cigarette smoke exposure can lead to the release of mitochondrial DNA (25) from epithelial cells, which activates ZBP1 and promotes the release of exosomes, contributing to disease progression (16). Additionally, decreased SIRT1 activity has been observed in COPD patients, potentially impairing cellular resilience to inflammation and oxidative stress (25, 26). Taken together, these findings suggest a potential regulatory role for SIRT1 in DC-derived exosome-mediated immune modulation during cigarette smoke exposure.

In light of these prior results, we hypothesized that SIRT1 may regulate the polarization of CD4^+^ T cells toward Th17 and Treg subsets via DC-derived exosomes in a manner linked to the autophagy and necroptosis pathways. The goal of this study was therefore to elucidate the underlying molecular mechanisms by which SIRT1 modulates immune responses in COPD, with a particular focus on the roles of autophagy, necroptosis, and exosome-mediated T cell polarization. Through this investigation, we aim to contribute to the understanding of COPD immunopathogenesis and identify novel targets for therapeutic intervention.

## Materials and methods

### Reagents

Recombinant murine granulocyte-macrophage colony-stimulating factor (GM-CSF) and recombinant murine IL-4 were obtained from PeproTech (London, UK). The SIRT1 activator SRT1720 was purchased from MedChemexpress (MedChemexpress Biotechnology Company, USA). CD86 antibody was acquired from Affinity Biosciences LTD (Changzhou, China). SybrGreen qPCR Master Mix (2×) was obtained from Aidlab Biotechnologies Co., Ltd. (Beijing, China). The CD11c Magnetic Bead Sorting Kit, CD4^+^ T Cell Magnetic Bead Sorting Kit, and antibodies specific for CD11c/PE, CD4/FITC, CD25/PE, FOXP3/APC, and IL-17A/APC were obtained from BD Pharmingen (San Diego, CA, USA). Antibodies for SIRT1, Atg16L1, p62/SQSTM1(p62), LC3-II, CD63, HSP70, CD81, ZBP1, RIPK3, MLKL, Caspase-8, and Caspase-3 were purchased from Abcam (Cambridge, UK). ELISA kits for IL-17A, IL-17F, IL-10, and IL-4 were also obtained from Abcam (Cambridge, UK). Primers for the genes encoding Sirt1, Atg16l1, RORγt, Foxp3, Il17a, IL-17F, IL-10, and IL-4, specific to C57BL/6J mice, were purchased from Sangon Biotech Co., Ltd. (Shanghai, China).

### Experimental animals

C57BL/6J mice (4–6 weeks old) were obtained from Changzhou Cavens Laboratory Animal Co., Ltd. (Changzhou, China). All animal experiments were approved by the Animal Ethics Committee of Guangxi Medical University.

### Preparation of cigarette smoke extract

CSE was prepared following the method described by Liu et al. (10, 27). In brief, smoke from cigarettes without filters was drawn into 20 mL of serum-free DMEM using a negative-pressure suction device. The solution was adjusted to pH 7.4 with 1 mol/L NaOH and filtered through a 0.22 μm filter to yield 100% CSE. Commercial Liusanjie cigarettes (Guangxi Tobacco Industry Co., Ltd.; tar: 6 mg; nicotine: 0.6 mg; CO: 5 mg) were burned until 0.5 cm above the filter remained. For 10% CSE, smoke from two cigarettes was bubbled into 20 mL of serum-free RPMI medium at a rate of one cigarette per minute. The pH was adjusted to 7.4, and the optical density was measured at 350 nm (0.81 ± 0.03). The solution was then sterilized using a 0.45 μm filter cartridge (25 mm Acrodisc; Pall, Ann Arbor, MI). CSE was freshly prepared on the day of each experiment. The absorbance at 320 nm showed minimal variation between preparations. Control medium was generated by bubbling air through 20 mL of serum-free RPMI (pH 7.4), followed by identical filtration.

### Bone marrow-derived DC culture

**Figure 1:**
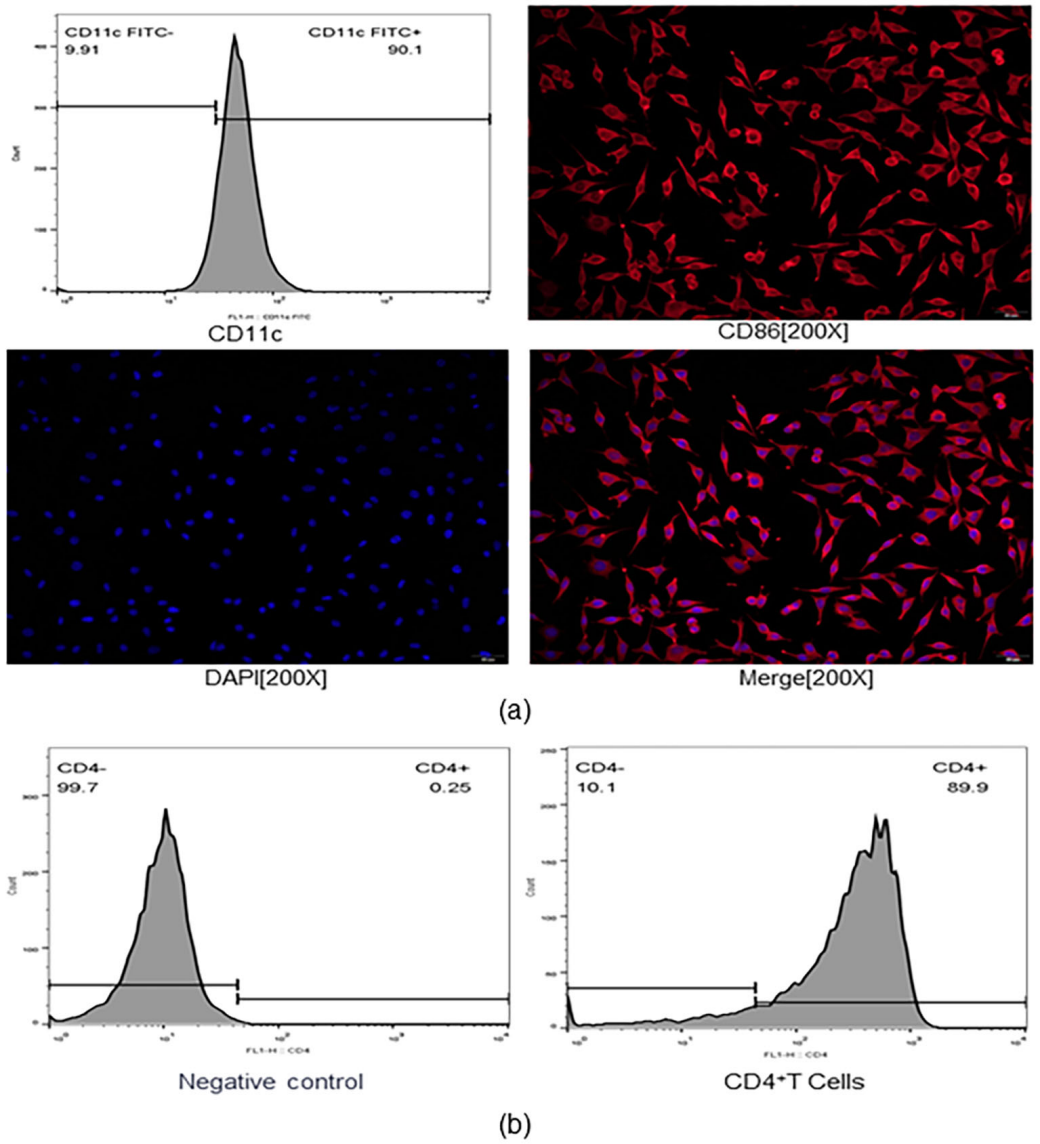
**(a)** Over 90% of cells exhibited high CD11c expression, with similarly high levels of CD86 expression. **(b)** CD4^+^ cell purity rates were as high as 89%.

Bone marrow-derived DCs were generated using the method described by Inaba and Kim et al. (10, 28, 29). Briefly, the femur and tibia were transferred to a new sterile culture dish containing phosphate-buffered saline (PBS). The bone marrow was exposed by careful dissection using sterile scissors. The bone marrow cavity was then flushed repeatedly with 2 mL of PBS using a syringe to collect all bone marrow cells, continuing until the bones appeared white. The resulting cell suspension was gently mixed in the presence of additional PBS and centrifuged at 1,500 rpm for 10 minutes at room temperature. The supernatant was carefully aspirated, and 5 mL of lymphocyte separation medium was added to a sterile centrifuge tube. The cell suspension was layered onto the surface of the separation medium and centrifuged at 1,500 rpm for 20 minutes at room temperature. Following centrifugation, three distinct layers were observed: the upper layer consisted primarily of PBS, the lower layer contained red blood cells and granulocytes, and the middle layer comprised the lymphocyte separation medium. A narrow, cloudy white band—enriched in mononuclear cells—was visible at the interface between the upper and middle layers. This interfacial cell layer was carefully collected using a pipette and transferred to a sterile centrifuge tube. PBS was added, and the cells were gently resuspended and centrifuged again at 1,500 rpm for 10 minutes at room temperature. The supernatant was discarded, and the washing step was repeated with PBS followed by centrifugation at 1,000 rpm for 5 minutes. The final cell pellet was resuspended in Red Blood Cell Lysis Buffer. After red blood cell lysis, the cells were mixed with PBS, and centrifuged at 1,000 rpm for 5 minutes. After removing the suspension, collect the cells, which are the mononuclear cells. The mononuclear cells were cultured at a density of 1 × 10^6^ cells/mL in RPMI-1640 supplemented with GM-CSF (40 ng/mL) and IL-4 (10 ng/mL). The medium was replaced every two days. On the sixth day, following resuspension of the cells in an appropriate volume of phosphate-buffered saline (PBS), the cell suspension was collected and centrifuged at 1,500 rpm for 10 minutes at room temperature. The supernatant was then carefully removed and discarded. The cells were harvested and purified using a CD11c magnetic bead sorting kit. For immunofluorescence staining, adherent cells on slides were washed three times with PBS (3 minutes each), fixed with 4% paraformaldehyde for 15 minutes, and washed again. Cells were permeabilized with 0.5% Triton X-100 for 20 minutes at room temperature, followed by additional PBS washes. After blotting excess liquid, slides were blocked with normal goat serum for 30 minutes at room temperature. Excess serum was blotted off, and primary antibody (anti-CD86) was added. Slides were incubated overnight at 4 °C in a humidified chamber. The next day, slides were washed, incubated with Cy3-labeled goat anti-rabbit IgG for 1 hour in the dark at 20–37 °C, and washed again. Nuclei were stained with DAPI for 5 minutes in the dark, followed by four PBS washes. Slides were mounted with anti-fade medium and observed using a fluorescence microscope. Flow cytometry confirmed that the DCs were suitable for downstream assays (The purity of dendritic cells exceeded 90%, **Figures 1a**).

### Cell treatment

DCs were prepared as described in prior reports (10, 28, 29). Cells were serum-starved and exposed to 1.5% CSE for 24 hours (10, 27). After washing, DCs were treated with purified SRT1720 (30, 31). Control DCs were treated with SRT1720 without CSE exposure. All DCs were then harvested for subsequent analysis.

### Exploration of the relationship of SIRT1 to autophagy and necroptosis

To explore the relationship between SIRT1 expression, autophagy, and necroptosis, DCs were serum-starved and either exposed or unexposed to 1.5% CSE for 24 hours (10, 27). Post-wash, cells were treated with the SIRT1 agonist SRT1720 (30, 31). Control cells received SRT1720 without CSE exposure. Cells were plated in 6-well plates at a density of 5 × 10^5^ cells/well. After 24 hours, 5% CSE and 10 μM SRT1720 were added, and cultures were maintained for 36 hours before cell collection for Western blotting (30, 31).

### Western blotting

DCs were generated from bone marrow-derived mononuclear cells isolated from C57BL/6J mice and divided into four groups: DC group, SRT1720 treated group, CSE-exposed group and SRT1720-treated CSE-exposed DC group. After culture, the supernatant was discarded from the wells, and the cells were washed twice with 2 mL of PBS. Cells were then digested with 0.25% trypsin, and digestion was monitored until cells became rounded. The trypsin was aspirated, and 1 mL of complete culture medium was added. Cells were centrifuged at 1200 rpm for 3 minutes to collect the pellet (approximately 1 × 10^6^ cells). The pellet was resuspended in 200 μL of pre-chilled RIPA lysis buffer supplemented with PMSF (1:100 dilution), mixed thoroughly, and fully lysed. The lysate was then centrifuged at 12,000 rpm for 10 minutes, and the supernatant was collected, quantified, and stored at −80 °C. For protein quantification, a BCA working solution was prepared by mixing BCA reagents A and B at a 50:1 ratio. A 200 μL volume of the BCA working solution was added to each well of a microplate, which was then shaken for 30 seconds and incubated at 37 °C for 30 minutes. Absorbance was measured at 562 nm. A standard curve was generated using absorbance (Y-axis) and protein concentration (mg/mL, X-axis). Equal amounts of protein lysates (20 μg) were separated on 8–15% polyacrylamide gels and transferred to PVDF membranes. Membranes were blocked with 5% non-fat dry milk in PBS-T and incubated overnight at 4 °C with the primary antibodies specific for SIRT1, ZBP1, RIPK3, MLKL, Caspase-8, Caspase-3, ATG16L1, LC3B, and p62. After incubation, membranes were washed three times with TBST (1 minute per wash). Membranes were then incubated with HRP-conjugated secondary antibodies (1:5000) at 37 °C for 1 hour with gentle shaking, followed by three washes in TBST (5 minutes each). Protein bands were visualized using ECL substrate and imaged with a chemiluminescence detection system.

### Isolation of DC-derived exosomes

**Figure 2:**
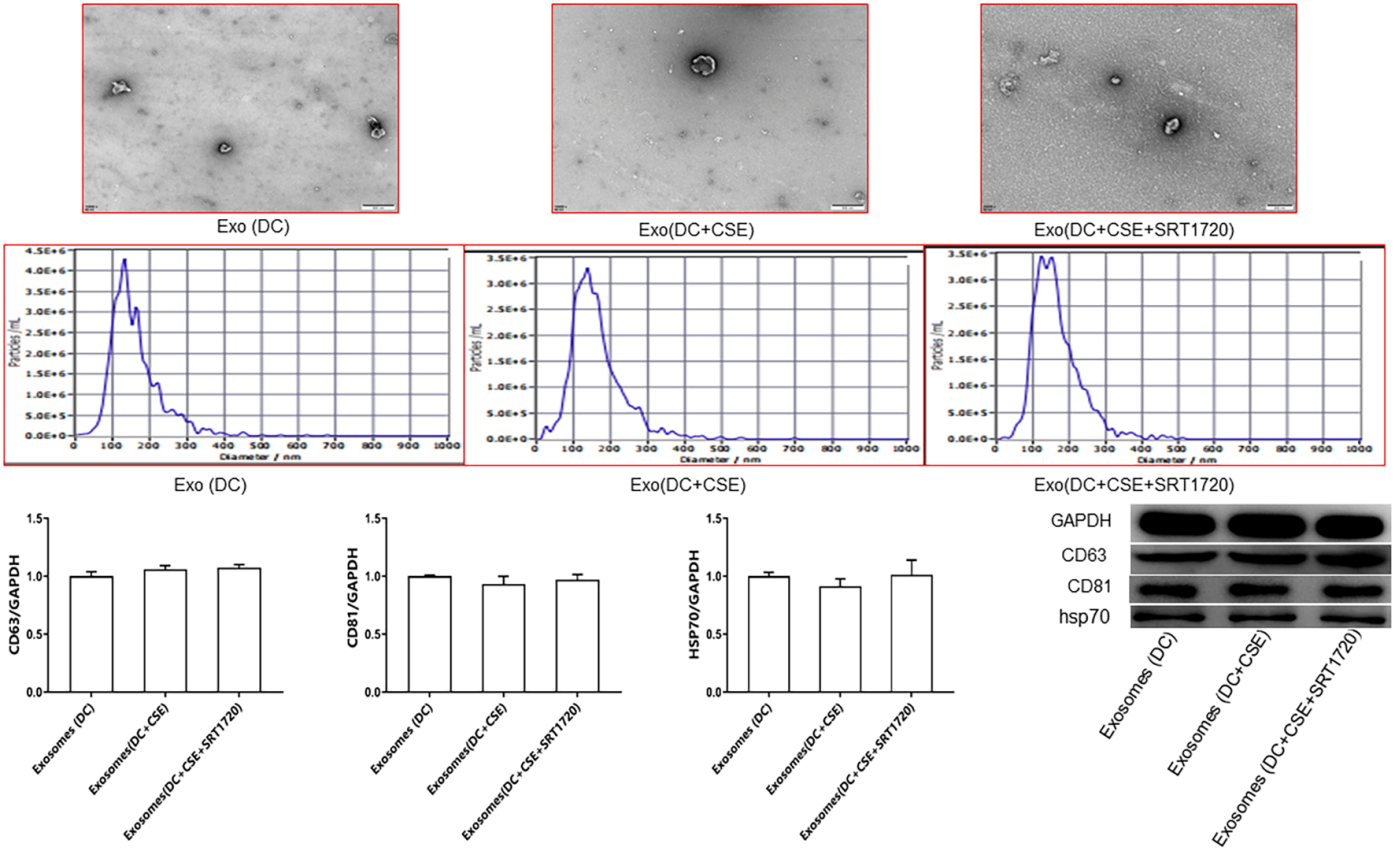
Exosomes observed via transmission electron microscopy were typical circular/ovoid vesicles surrounded by a double membrane. The distribution of exosome diameters as determined by nanoparticle tracking analysis is shown, demonstrating that particles were within the exosome size range (30 nm - 150 nm). Western blotting results revealing no significant difference in the protein levels of CD81, CD63, and HSP70 in exosomes cross groups (*p* > 0.05). Data are presented as the mean ± SD and were analyzed using ANOVA followed by the Newman−Keuls *post hoc* test for multiple comparisons.

DCs were cultured in their respective experimental groups. Once cell confluency reached approximately 70%, the culture medium was replaced with serum-free or exosome-depleted serum medium following three washes with sterile PBS. The culture supernatant was collected and placed into centrifuge tubes. Sequential centrifugation steps were performed at 4 °C: 300 × g for 10 minutes, 800 × g for 15 minutes, and 10,000 × g for 30 minutes to remove cells and debris. The resulting supernatant was transferred to a new tube and ultracentrifuged at 120,000 ×g for 70 minutes at 4°C. The pellet was resuspended in PBS and ultracentrifuged at 120,000 × g for 70 minutes (32). Exosomes were characterized by transmission electron microscopy (Hitachi, Japan) and nanoparticle tracking analysis (Matrix GmbH, Germany) (**Figure 2**). The protein markers CD81, CD63, and HSP70 were detected by Western blotting (32, 33), and there was not statistical significance among the three groups (control DCs, CSE-exposed DCs, and SRT1720-treated CSE-exposed DCs) (**Figure 2**). The final pellet was confirmed to be exosomes. Protein concentrations for DC-derived exosomes were measured using the BCA assay (34). After quantification, exosomes were stored at −80°C.

### CD4^+^ T cell isolation and identification

The method for isolating CD4^+^ T cells was based on previous studies by Liu et al. (10). Mice were euthanized by cervical dislocation, and spleens were aseptically removed and transferred into centrifuge tubes containing 5 mL of RPMI-1640 medium. In a sterile Petri dish, spleens were mechanically disrupted using sterile needles until the tissue became translucent, indicating cell release. Cell suspensions were gently pipetted ten times to break up clumps and then filtered into 15 mL Corning tubes. Cells were centrifuged at 500 × g for 4 minutes, and the supernatant was discarded. The pellet was resuspended in 2 mL of red blood cell lysis buffer and incubated on ice for 2 minutes. Lysis was neutralized with PBS containing BSA, followed by centrifugation and resuspension in PBS. Lymphocyte separation fluid was added to a new 15 mL sterile centrifuge tube. The cell suspension was carefully layered above the separation fluid at a 45° angle and centrifuged at 1500 rpm for 10 minutes. The lymphocyte layer was collected and resuspended in 4 mL of magnetic bead buffer. Cells were counted and centrifuged at 1500 rpm for 10 minutes. Based on the cell count, an appropriate volume of CD4 magnetic bead sorting antibody was added, and cells were incubated in the dark at 4 °C for 15 minutes. Cells were washed twice with buffer, centrifuged, and resuspended. The sorting column was rinsed with 500 μL of buffer three times. Positively sorted CD4^+^ cells were collected by eluting with buffer into a centrifuge tube. Cells were centrifuged at 1500 rpm for 10 minutes, and the pellet was resuspended for flow cytometric analysis. A total of 1 × 10^6^ single cells were resuspended in 100 μL of PBS and stained with flow cytometry antibodies. The mixture was incubated at 4 °C in the dark for 30 minutes, followed by two PBS washes and centrifugation at 1200 rpm for 10 minutes. The pellet was gently resuspended and adjusted to 300 μL for flow cytometric detection, confirming that CD4^+^ cells were suitable for subsequent experiments (The purity of CD4^+^ T cells exceeded 89%, **Figures 1b**).

### CCK-8 assay

**Figure 3:**
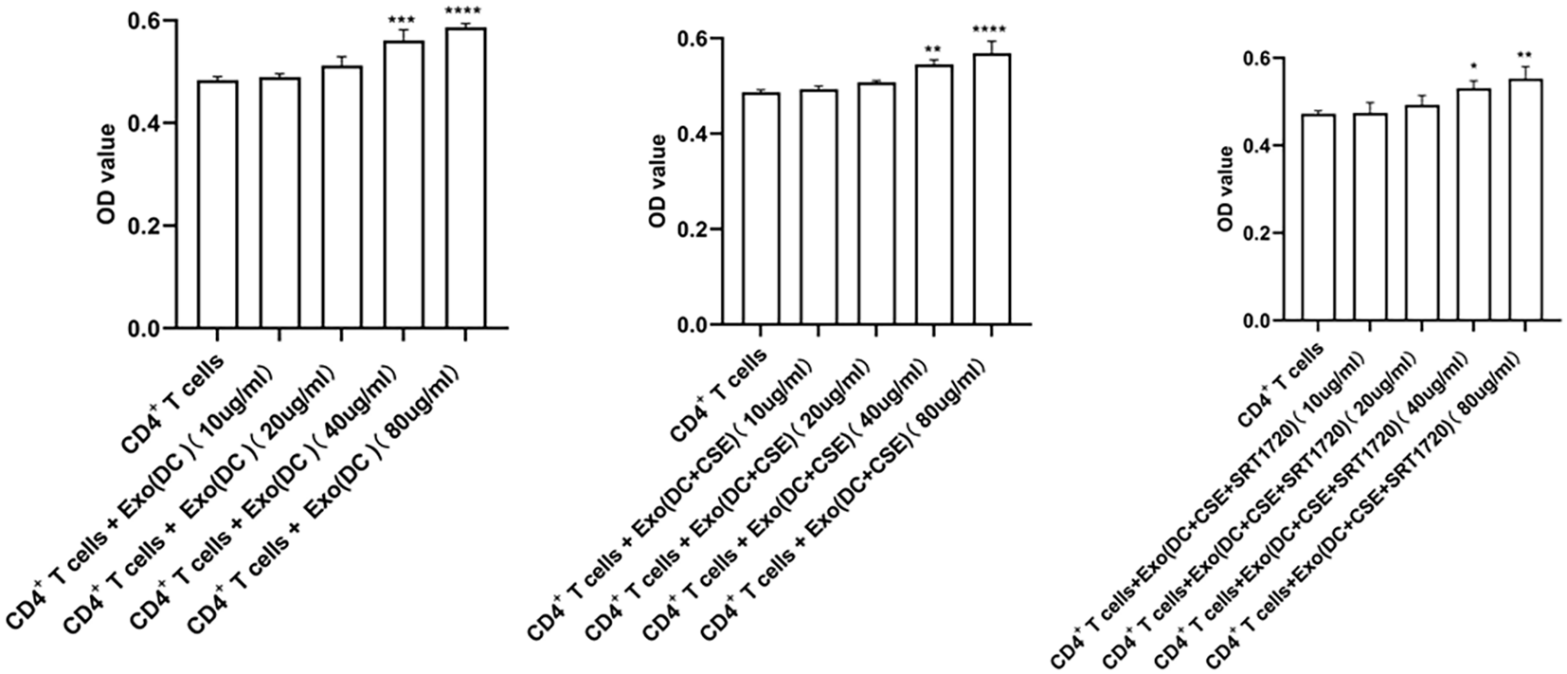
Comparison of proliferation showing a significant increase in CD4^+^T cells proliferation when treated with exosomes at 40 μg/mL relative to a 10 μg/ml concentration or 20 μg/ml concentration of exosomes in the DC group. *p < 0.05, **p < 0.01, ***p < 0.001, ****p < 0.0001. Data are represented as the mean ± SD and were analyzed using ANOVA followed by the Newman−Keuls *post hoc* test for multiple comparisons.

CD4^+^ T cells were divided into the following groups: CD4^+^ T cells alone; CD4^+^ T cells + DC-derived exosomes (10 μg/mL); CD4^+^ T cells + DC-derived exosomes (20 μg/mL); CD4^+^ T cells + DC-derived exosomes (40 μg/mL); CD4^+^ T cells + DC-derived exosomes (80 μg/mL); CD4^+^ T cells + exosomes from CSE-exposed DCs (10 μg/mL); CD4^+^ T cells + exosomes from CSE-exposed DCs (20 μg/mL); CD4^+^ T cells + exosomes from CSE-exposed DCs (40 μg/mL); CD4^+^ T cells + exosomes from CSE-exposed DCs (80 μg/mL); CD4^+^ T cells + SRT1720-treated, CSE-exposed DC-derived exosomes (10 μg/mL); CD4^+^ T cells + SRT1720-treated, CSE-exposed DC-derived exosomes (20 μg/mL); CD4^+^ T cells + SRT1720-treated, CSE-exposed DC-derived exosomes (40 μg/mL); and CD4^+^ T cells + SRT1720-treated, CSE-exposed DC-derived exosomes (80 μg/mL). CD4^+^ T cells were cultured and adjusted to a concentration of 5×10^4^ cells/mL. Cells were seeded in 96-well culture plates at 200 μL per well according to the group assignments. After the addition of exosomes as outlined above, cells were cultured for 48 hours. At the designated time point, cell morphology was observed under a microscope and photographed. Subsequently, 10 μL of CCK-8 reagent was added to each well and incubated at 37 °C in a 5% CO_2_ incubator for 2 hours in the dark. The optical density (OD) was measured at 450 nm using a microplate reader. The CCK-8 assay results showed that exosomes in all treatment groups promoted the proliferation of CD4^+^ T cells. Among the tested concentrations, a concentration of 40 μg/mL exosomes was selected for subsequent experiments (**Figure 3**).

### Mixed lymphocyte reaction

CD4^+^ T cells were isolated from the spleens of C57BL/6J mice using a magnetic bead-based cell sorting method according to the manufacturer’s instructions. The purified CD4^+^ T cells were used in mixed lymphocyte reaction (MLR) assays and divided into four experimental groups: CD4^+^ T cells alone; CD4^+^ T cells co-cultured with DC-derived exosomes; CD4^+^ T cells co-cultured with exosomes derived from cigarette smoke extract (CSE)-exposed dendritic cells (DCs); and CD4^+^ T cells co-cultured with exosomes from SRT1720-treated, CSE-exposed DCs. Cells were cultured with exosomes at a concentration of 40 μg/mL, a dose previously CCK-8 assay determined to effectively activate CD4^+^ T cells (**Figure 3**). The MLR was performed in round-bottom 96-well microplates with 0.2 mL of serum-free medium per well. After 24 hours of co-culture, cell activation and differentiation were assessed by quantifying mRNA expression levels of Sirt1, Atg16l1, RORγt, Foxp3, Il17a, IL-17F, IL-10, IL-4, and Gapdh via real-time quantitative PCR (RT-qPCR). Th17 and regulatory T cell (Treg) populations were further analyzed by flow cytometry. Culture supernatants were collected for measurement of cytokine levels.

### Flow cytometry

T cells were stained with APC-conjugated anti-CD4 and FITC-conjugated anti-TCR Vα2 or PE-conjugated anti-CD40L antibodies. For intracellular cytokine analysis, T cells were stimulated with 10^−7^ M phorbol-12-myristate-13-acetate (PMA), 1 μg/mL ionomycin, and 3 μM monensin for 4 hours. Cells were then fixed, permeabilized, and stained with FITC-labeled anti-CD4 and PE-labeled anti-IL-17A antibodies. BD Cytofix/Cytoperm buffer was used for permeabilization. Staining was performed on ice for 30 minutes, followed by washing with ice-cold phosphate-buffered saline containing 0.1% sodium azide (NaN_3_) and 0.5% bovine serum albumin (Sigma-Aldrich). Samples were analyzed using a BD FACSCalibur flow cytometer. Mean fluorescence intensities were calculated by subtracting background fluorescence from isotype controls. Data were analyzed with CellQuest software (BD Pharmingen). FoxP3 intracellular staining was conducted using directly conjugated antibodies and the eBioscience Fixation/Permeabilization Buffer system, in accordance with the manufacturer’s instructions. CD4 surface staining was performed prior to permeabilization using PE- or FITC-conjugated antibodies. Isotype controls were used for compensation and to confirm antibody specificity. Flow cytometry analysis was completed using FCS Express V4.

### Gene expression analysis

Total RNA was isolated from MLR samples using the RNeasy Kit (Qiagen, Düsseldorf, Germany). First-strand cDNA synthesis was performed using 1 μg of total RNA and SuperScript II Reverse Transcriptase (Invitrogen). PCR amplification of the genes encoding Sirt1, Atg16l1, RORγt, Foxp3, Il17a, IL-17F, IL-10, IL-4, and Gapdh was carried out with the specific primers listed in **Table 1**.

**Table 1:** Primers used for real-time quantitative PCR analysis.

Genes	Primer	Sequence (50→30)
Sirt1	Forward primer	CACCTCTTCATATTTCGG
	Reverse primer	CATTGTTGTTTGTTGCTT
Atg16l1	Forward primer	TCTCTTCCATCCCAGTCCCC
	Reverse primer	GCTTCCCAAAGTTTCACCCT
RORγt	Forward primer	GAAAGCAGGAGCAATGGA
	Reverse primer	GCTGAGGAAGTGGGAAAA
Foxp3	Forward primer	CCTCCCACCACCTTCTGC
	Reverse primer	GCCTTGCCTTTCTCATCC
Il17a	Forward primer	CACTGAGGCCAAGGACTT
	Reverse primer	CGTGGAACGGTTGAGGTA
IL-17F	Forward primer	GAATCTTCAACCAAAACCAGGGCA
	Reverse primer	CAGGATTTCTTGCTGAATGGCGAC
IL-10	Forward primer	ATGCAGGACTTTAAGGGT
	Reverse primer	TTATTTTCACAGGGGAGA
IL-4	Forward primer	TCCTGCTCTTCTTTCTCG
	Reverse primer	GTTTGGCACATCCATCTC
Gapdh	Forward primer	GGAGCGAGATCCCTCCAAAAT
	Reverse primer	GGCTGTTGTCATACTTCTCATGG

### RNA isolation and real-time PCR

Total RNA was extracted from T cells in the MLR assays using TRIzol reagent (Carlsbad, CA, USA), following standard protocols. Reverse transcription was performed using SuperScript II. Real-time PCR was carried out using SYBR Green on an ABI Prism 7500 Sequence Detection System (Applied Biosystems, Foster City, CA, USA) to assess the expression levels of genes encoding Sirt1, Atg16l1, RORγt, Foxp3, Il7a, IL-17F, IL-10, IL-4, and Gapdh. The thermocycler conditions were as follows: 50 °C for 2 minutes, 95 °C for 10 minutes, followed by 40 cycles of 95 °C for 15 seconds and 60 °C for 1 minute. The Ct values were automatically generated and validated based on melting curve analysis. Expression differences were calculated using the 2^-ΔΔCt^ method.

### ELISAs

Cytokine levels (IL-17A, IL-17F, IL-10, and IL-4) in the culture supernatants were measured using ELISA kits specific for mouse cytokines (ExCell Bio, China), in accordance with the manufacturer’s protocol.

### Statistical analysis

Data are expressed as means ± standard deviation (SD). Statistical significance was assessed using unpaired two-tailed Student’s t-tests or one-way analyses of variance (ANOVAs) followed by the Newman-Keuls *post hoc* test for multiple comparisons. All statistical analyses were performed using SPSS 27.0. A P-value < 0.05 was considered significant. The statistical figures were created using GraphPad Prism 8.

## Results

### Generation and identification of bone marrow-derived DCs and CD4^+^ T cells

Flow cytometry analysis revealed that 90.1% of CD11c*^+^* cells were present, and the DCs expressed high levels of CD86A (**Figure 1a**). The experimental results also indicated that 89.9% of CD4^+^ T cells were positive (**Figure 1b**), indicating that subsequent experiments could be carried out.

### DC-derived exosome isolation

Transmission electron microscopy revealed that the isolated exosomes appeared as typical circular or oval vesicles surrounded by a double membrane. Nanoparticle tracking analysis showed that the particle size distribution of the exosomes ranged between 30 nm and 150 nm, which is consistent with the known size range of exosomes. Western blotting indicated no significant differences in the expression of exosomal markers CD81, CD63, and HSP70 among the different groups (*p* > 0.05) (**Figure 2**).

### Selection of optimal exosome concentrations for MLR assays

CCK-8 assay results showed that exosomes from all groups promoted CD4^+^ T cell proliferation. Among the tested concentrations, 40 μg/mL produced a significantly stronger proliferative effect. Therefore, 40 μg/mL was selected as the optimal concentration for subsequent experiments (**Figure 3**).

### Evaluation of SIRT1 protein levels in CSE-exposed DCs

**Figure 4:**
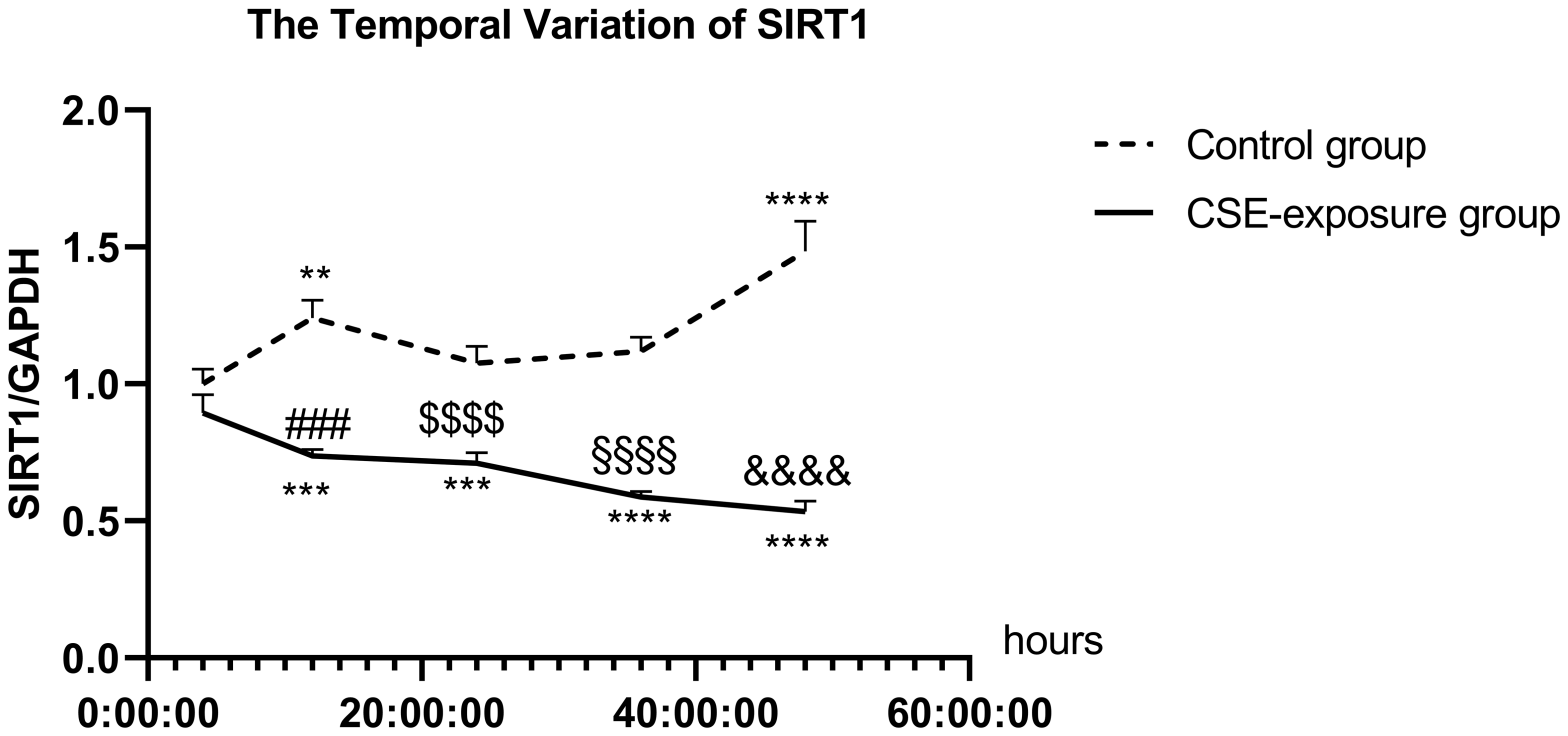
The expression of SIRT1 in dendritic cells (DCs) was significantly reduced following cigarette smoke treatment when compared with the respective control groups at various time points. Specifically, SIRT1 expression in the CSE-treated DC group was reduced at 12 h, 24 h, 36 h, and 48 h compared to that in the untreated DC group at the corresponding time points (**Comparison with the DC 4 h group, p< 0.01; ***Comparison with the DC 4 h group, *p* < 0.001; ****Comparison with the DC 4 h group, *p* < 0.0001; ###Comparison with the DC 12 h group, *p* < 0.001; $$$$Comparison with the DC 24 h group, *p* < 0.0001; §§§§Comparison with the DC 36 h group, *p* < 0.0001; &&&&Comparison with the DC 48 h group, *p* < 0.0001). For western blot analyses, values are given as mean ± SD of at least three independent experiments (n = 3), and the results were analyzed using unpaired two-tailed Student’s t-tests.

Western blotting demonstrated that SIRT1 expression in DCs exposed to CSE was significantly downregulated at all time points compared to the corresponding control groups. Specifically, SIRT1 expression decreased at 4, 12, 24, 36, and 48 hours post-CSE exposure relative to baseline (**Figure 4**).

### Analyses of protein levels of autophagy and necroptosis in DCs

**Figure 5:**
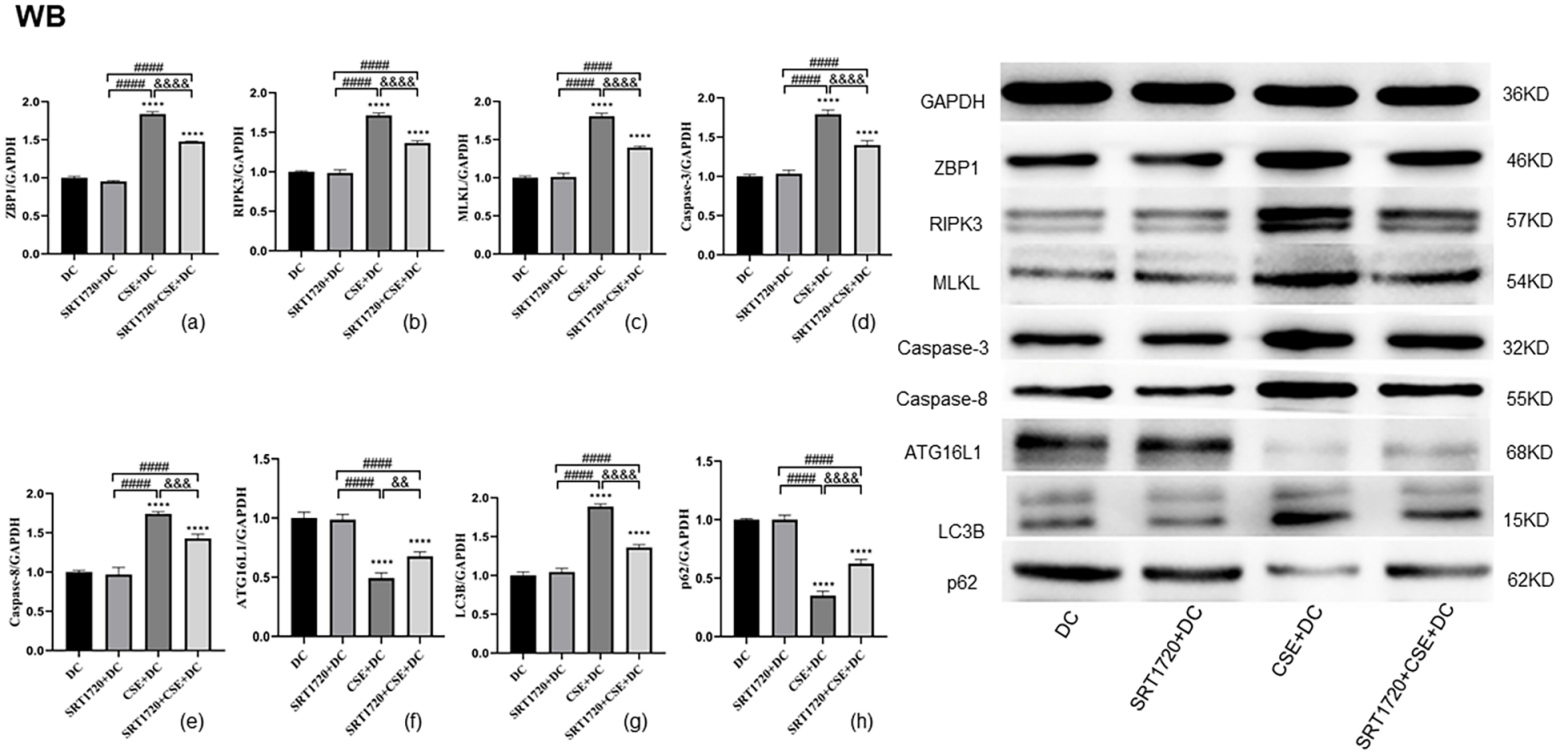
Data from western blot experiments are given as the mean ± SD of at least three independent experiments (n = 3) for panels **(a–h)**. Compared with the DC group, ****p < 0.0001. Compared with the SRT1720 + DC group, ####*p* < 0.0001. Compared with the CSE + DC group, &&*p* < 0.01, &&&*p* < 0.001, &&&&*p* < 0.0001. Statistical comparisons were performed using ANOVA followed by the Newman−Keuls post hoc test for multiple comparisons.

Western blotting revealed that, compared to the control DC group, treatment with SRT1720 alone did not significantly alter the protein levels of ZBP1, RIPK3, MLKL, Caspase-8, Caspase-3, ATG16L1, LC3B, or p62 (**Figure 5**). In contrast, DCs exposed to CSE showed significantly increased expression of the necroptosis-related proteins ZBP1, RIPK3, MLKL, Caspase-8, and Caspase-3 (**Figure 5**), along with a significant reduction in levels of the autophagy-related proteins ATG16L1 and p62 (**Figure 5**). LC3B expression was also significantly elevated (**Figure 5**). However, in DCs exposed to CSE and treated with SRT1720, the expression of necroptosis-related proteins was significantly decreased, while autophagy-related proteins ATG16L1 and p62 were significantly more abundant, and LC3B expression was reduced (**Figure 5**).

### Analyses of Th17 and Treg populations

**Figure 6:**
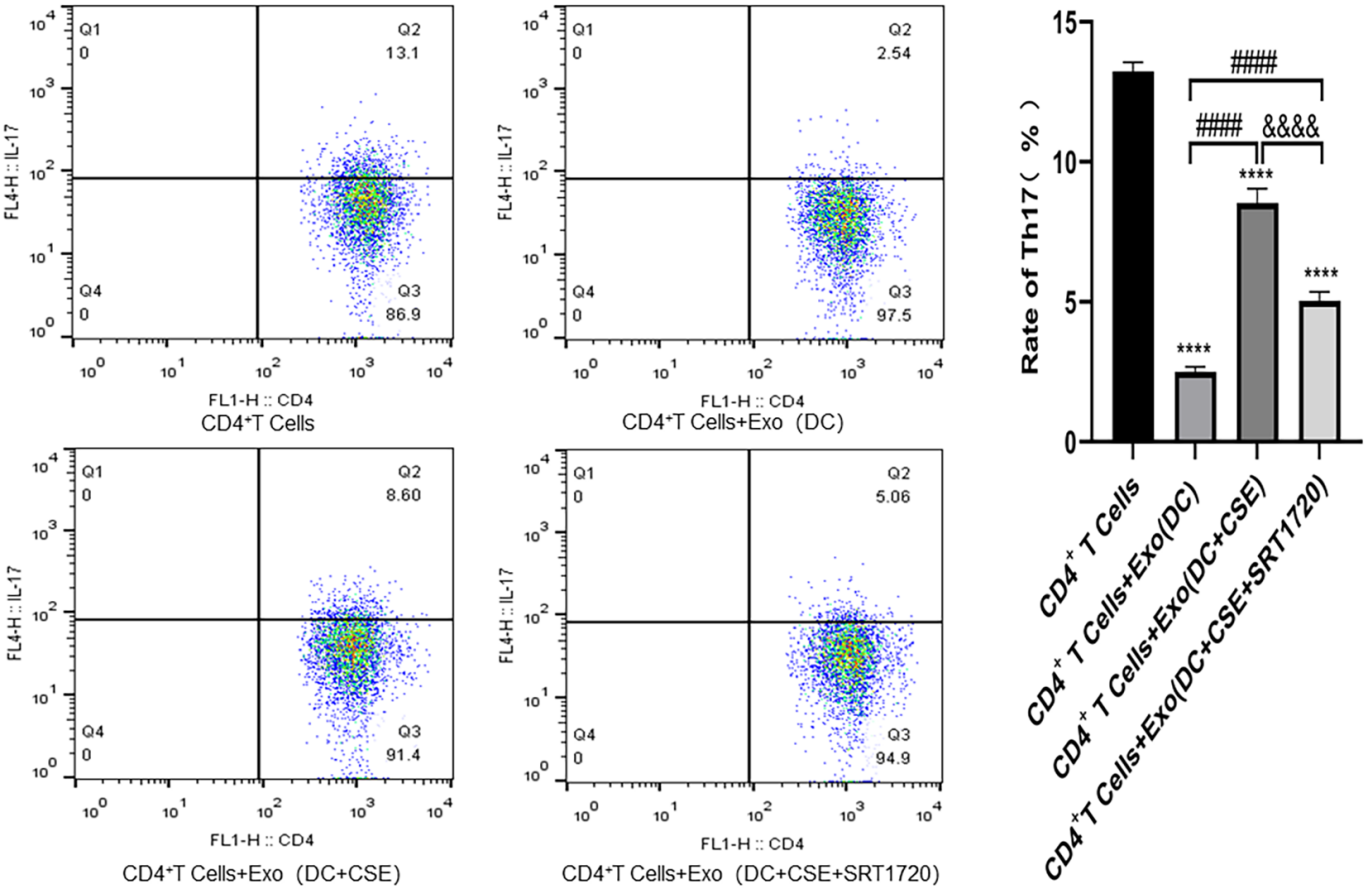
Flow cytometry data are given as the mean ± SD of at least three independent experiments (n = 3). Compared with CD4^+^ T cells: ****p < 0.0001. Compared with CD4^+^ T cells + exosomes (DC group): ####*p* < 0.0001; Compared with CD4+ T cells + exosomes (DC +CSE) group: &&&&p < 0.0001). Statistical comparisons were performed using ANOVA followed by the Newman−Keuls *post hoc* test for multiple comparisons.

**Figure 7:**
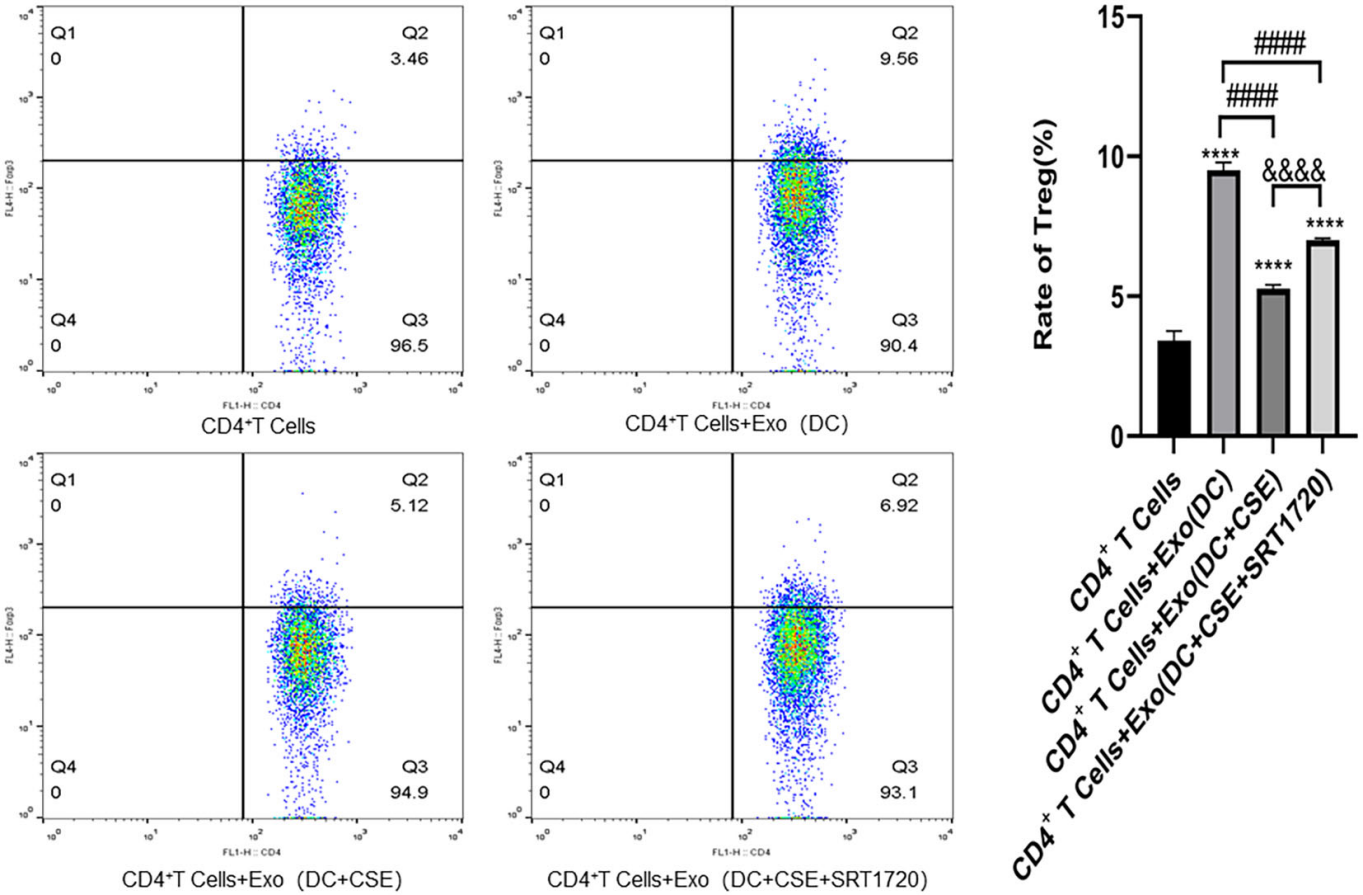
For flow cytometry data, values are given as the mean ± SD of at least three independent experiments (n = 3). Compared with CD4^+^ T cells: ****p < 0.0001; Compared with CD4^+^ T cells + exosomes (DC group): ####*p* < 0.0001; Compared with CD4+ T cells + exosomes (DC+CSE) group: &&&&p < 0.0001. Statistical comparisons were performed using ANOVA followed by the Newman−Keuls *post hoc* test for multiple comparisons.

Flow cytometry and RT-qPCR analyses showed that, compared with the CD4^+^ T cell blank control group, the frequency of Th17 cells were decreased, whereas Treg cell frequencies were increased in the DC-derived exosomes (DCexos) co-culture group. In contrast, co-culture with exosomes from CSE-exposed DCs resulted in increased Th17 frequency along with reduced Treg frequency. Treatment with DCexos from SRT1720 in the CSE-exposed group decreased Th17 frequencies but increased Treg frequencies (**Figure 6**, **7**).

*Sirt1, Atg16l1, RORγt, Foxp3, and Cytokine mRNA levels in CD4^+^ T cells.*

**Figure 8:**
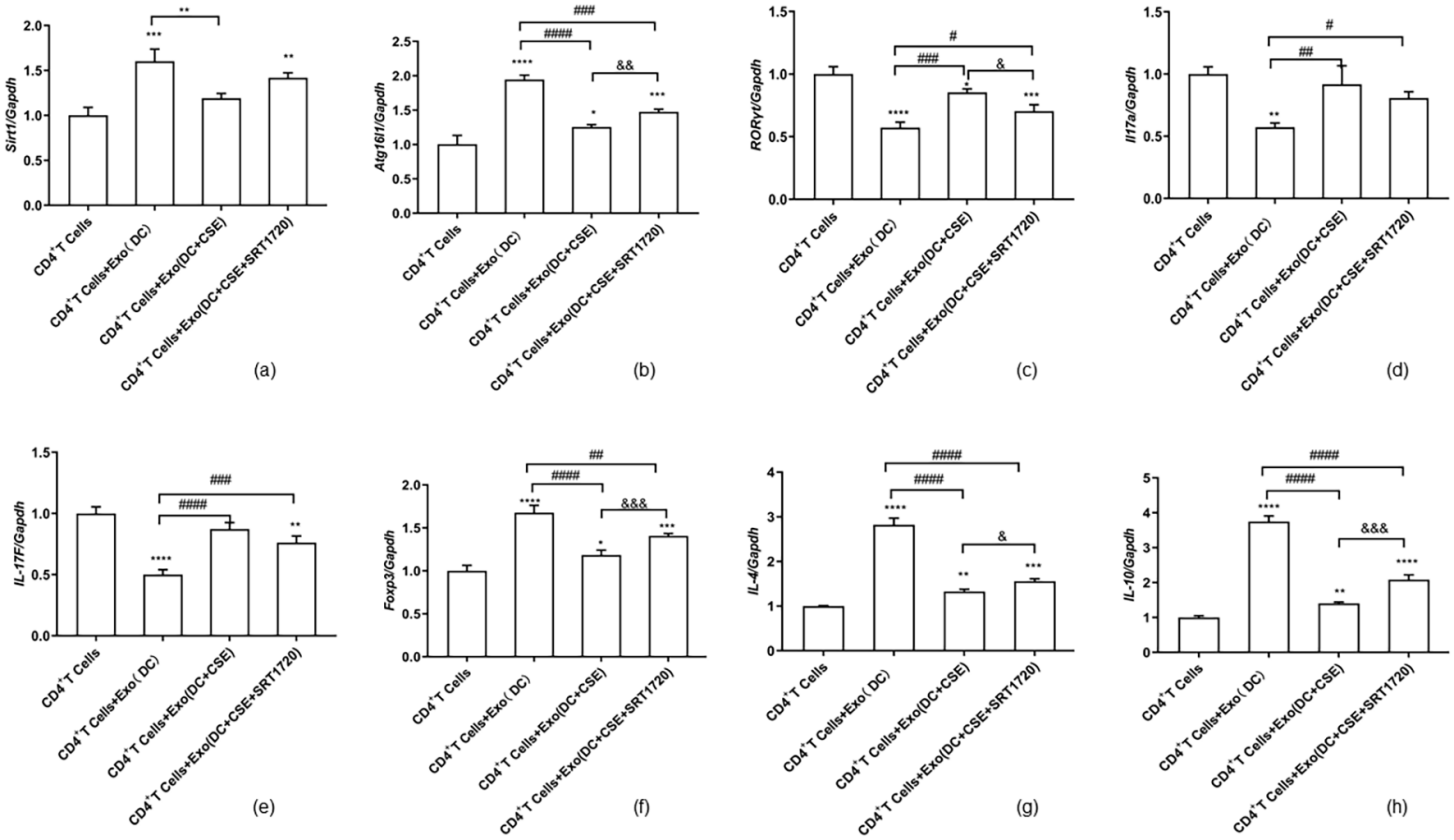
RT-qPCR data are given as the mean ± SD of at least three independent experiments (n = 3) for panels **(a–h)**. Compared with the CD4^+^ T cell group, *p < 0.05, **p < 0.01, ***p < 0.001, ****p < 0.0001. Compared with the CD4^+^ T cells + exosome group, #*p* < 0.05, ##*p* < 0.01, ###*p* < 0.001, ####*p* < 0.0001. Compared with CD4^+^ T cells + exosomes (DC+CSE) group: &*p* < 0.05, &&*p* < 0.01, &&&*p* < 0.001. Statistical comparisons were performed using ANOVA followed by the Newman−Keuls post hoc test for multiple comparisons. Data are presented as the mean ± SD.

RT-qPCR results indicated that, compared with the CD4^+^ T cell control group (blank group), the mRNA expression of Sirt1 and Atg16l1 was significantly increased when CD4^+^ T cells were co-cultured with DCexos (DC group). However, co-culturing CD4^+^ T cells with exosomes derived from CSE-exposed DCs resulted in significantly decreased the mRNA-level expression of Sirt1 and Atg16l1. Treatment of these cells with SRT1720 significantly upregulated the expression of both Sirt1 and Atg16l1 compared to the group without SRT1720 treatment. Compared to CD4^+^T cells in the blank control group, the expression levels of RORγt, Il17a, and IL-17F mRNA were downregulated, while those of Foxp3, IL-4, and IL-10 mRNA were upregulated in the CD4^+^T cells co-cultured with dendritic cell-derived exosomes (DC group). The expression levels of RORγt, Il17a, and IL-17F mRNA increased, while Foxp3, IL-4, and IL-10 mRNA expressions decreased, when CD4^+^ T cells were co-cultured with exosomes derived from CSE-exposed DCs. Furthermore, compared to the co-culture of CD4^+^T cells with exosomes derived from CSE-exposed DCs, the administration of SRT1720 resulted in a decrease in RORγt, Il17a, and IL-17F mRNA expression, while Foxp3, IL-4, and IL-10 mRNA expression increased after treatment with SRT1720 (**Figure 8**).

### Cytokine expression analyses by ELISA

**Figure 9:**
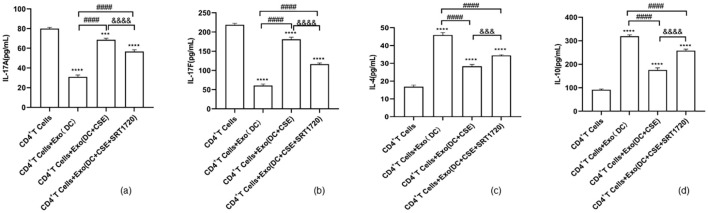
ELISA data are given as the mean ± SD of at least three independent experiments (n = 3) for panels **(a–d)**. Compared with CD4^+^ T cells: ***p < 0.001, ****p < 0.0001. Compared with CD4^+^ T cells + exosomes (DC group): ####*p* < 0.0001. Compared with CD4^+^ T cells + exosomes (DC+CSE) group: &&&*p* < 0.001, &&&&*p* < 0.0001. Statistical comparisons were performed using ANOVA followed by the Newman−Keuls post hoc test for multiple comparisons.

ELISA results demonstrated that, compared to the blank CD4^+^ T cell control group, the expression of IL-17A and IL-17F were significantly reduced, while the levels of IL-10 and IL-4 were significantly increased in CD4^+^ T cells co-cultured with DCexos (DC group). In the group co-cultured with exosomes derived from CSE-exposed DC, IL-17A and IL-17F levels were significantly increased, whereas IL-10 and IL-4 levels were significantly decreased compared to the DC group. Notably, treatment with SRT1720 reversed these changes, resulting in decreased IL-17A and IL-17F expression as well as increased IL-10 and IL-4 expression (**Figure 9**).

## Discussion

In this study, Th17 cell frequencies were found to decrease among CD4^+^ T cells cultured in an MLR assay together with DCexos, whereas, exposure to CSE led to an increase in Th17 cells. Conversely, Treg populations became more frequent in the presence of DCexos but decreased following CSE exposure. Elashiry et al. (13) demonstrated that DCs can regulate the differentiation of CD4^+^ T cells into Th17 and Treg subsets through the release of distinct exosomes, thereby playing a crucial role in maintaining the Th17/Treg balance. Russell et al. (16) further showed that cigarette smoke-induced extracellular vesicles from DCs promote the polarization of CD4^+^ T cells toward Th17 and Th1 phenotypes.

Exosomes are small extracellular vesicles secreted by various cell types and contain proteins, lipids, and RNAs, including miRNAs, mRNAs, and long non-coding RNAs (lncRNAs), that participate in numerous biological and pathological processes (13–15, 35). Recent studies have confirmed that cigarette smoke exposure alters lncRNA expression profiles, often resulting in their upregulation (36, 37). Certain lncRNAs play key roles in T cell development and function, influencing activation and differentiation through modulation of cytokine expression and signaling pathways. In the context of Th17 and Treg differentiation, specific lncRNAs may either promote or inhibit subset-specific differentiation by regulating the activity and expression of transcription factors (35–38). The differential expression patterns of miRNAs in activated and tolerogenic human DCs exhibit distinct characteristics, whichcontribute to regulating the Th17/Treg balance (39–41), and several miRNAs are upregulated following cigarette smoke exposure, contributing to the pathogenesis of COPD (41, 42). Moreover, lncRNAs can modulate the expression of downstream target genes by competitively binding to miRNAs, thereby affecting immune responses and T cell function (37, 43). Wu et al. (44) reported that among 437 lncRNAs expressed in immature dendritic cells (iDCs) and lipopolysaccharide-stimulated mature dendritic cells (mDCs), 87 were significantly upregulated in exosomes derived from mDCs, while 21 were downregulated. Bioinformatics analysis revealed that upregulated lncRNAs were closely associated with immune system processes, while downregulated lncRNAs were related to poly(A) RNA binding functions. Gene enrichment analysis further indicated that specific lncRNAs such as Procr-203, Clec4e-202, and Traf1–203 were highly correlated with immune regulatory functions, including T helper cell differentiation and the JAK-STAT signaling pathway. These findings suggest that exosomal lncRNAs derived from DCs may serve critical roles in immune regulation. At the same time, the co-culturing CD4^+^ T cells with CSE-exposed DCexos resulted in significantly decreased the mRNA-level expression of Sirt1 and Atg16l1. Recent studies have showed that lncRNA induced imbalance of Treg/Th17 involved with Sirt1 expression (15, 45, 46). Several studies have shown that the autophagy gene ATG16l1 plays an important role in the differentiation of T cells has documented that Selective deletion of ATG16l1 led to decrease of Treg in intestinal inflammation as well (14, 47, 48). What the mechanism of DC-derived exosomes differentiating CD4+T cells maybe need more investigation, which exosomes derived from DC under CSE-exposure and treating SIRT1 activator.

Here, SIRT1 levels in DCs were found to be significantly reduced following CSE exposure, with expression levels progressively decreasing over time. This reduction was accompanied by a shift in the Th17/Treg balance, induced by DCexos. However, treatment with a SIRT1 activator (SRT1720) reversed these effects, leading to decreased Th17 differentiation and increased Treg polarization mediated by DCexos under CSE exposure. Several studies have reported that downregulation of SIRT1 in DCs may alter both the release and functional properties of exosomes following cigarette smoke exposure (18, 25, 26, 46–48). Lee et al. (49) demonstrated that SIRT1 deficiency increases exosome secretion and is associated with the formation of enlarged LAMP1-positive lysosomes. The absence of SIRT1 not only affects lysosomal function but also modifies the composition of exosomes, resulting in the enrichment of ubiquitinated proteins (18, 46, 47). Taken together, current evidence suggests that DCexos play a significant role in modulating CD4^+^ T cell polarization toward Th17 and Treg subsets in the context of cigarette smoke exposure. Previous studies have established that DCs regulate the Th17/Treg balance through exosome-mediated transfer of microRNAs, cytokines, and other signaling molecules (7, 8, 10–12, 39, 40, 45). In this study, we observed that following CSE exposure, DCexos promoted Th17 polarization while suppressing Treg differentiation, leading to a disruption of the Th17/Treg balance. These findings are consistent with prior reports indicating that cigarette smoke disrupts immune tolerance and enhances pro-inflammatory responses in the lungs. Notably, our results also demonstrate that SIRT1 expression is downregulated under CSE exposure, contributing to the observed Th17/Treg imbalance. As a key regulator of inflammation and oxidative stress, SIRT1 is known to promote anti-inflammatory and immunosuppressive responses by enhancing Treg differentiation. Our data suggest that reduced SIRT1 expression under CSE exposure may impair the ability of DCexos to regulate CD4^+^ T cell polarization, although the precise mechanisms remain to be clarified.

In this study, SIRT1 expression was found to decline following CSE exposure, with concurrent increases in LC3B expression and reduced ATG16L1 and P62 levels on exposure to CSE. In addition, ZBP1, RIPK3, MLKL, Caspase-8, and Caspase-3 levels rose in response to CSE exposure. Importantly, the downregulation of ATG16L1 and P62/SQSTM1 in DCs induced by CSE was reversed by treatment with the SIRT1 activator SRT1720. Similarly, SIRT1 activation attenuated the CSE-induced increase in LC3B expression. Moreover, SIRT1 activation effectively suppressed the upregulation of necroptosis-related proteins ZBP1, RIPK3, MLKL, Caspase-8, and Caspase-3 in DCs. These results suggest that SRT1720 can mitigate CSE-induced autophagy dysfunction and necroptosis in DCs. Additionally, exosomes derived from DCs exposed to CSE promoted CD4^+^ T cell polarization toward the Th17 lineage and inhibited their differentiation into Tregs. This effect was significantly reduced following SIRT1 activation with SRT1720.

Cigarette smoke exposure has been shown to lead to a reduction in SIRT1 expression, which may be further exacerbated by dysregulated autophagy (25, 26, 48). Zhang et al. (48) showed that CSE exposure leads to DNA damage and a reduced NAD+/NADH ratio, thereby inhibiting SIRT1 activity. Activation of SIRT1 has been shown to alleviate aging-related phenotypes through autophagy-dependent mechanisms. SIRT1 deficiency not only impairs lysosomal function but also alters both the secretion and composition of exosomes. Notably, exosomes derived from SIRT1-deficient cells are enriched in ubiquitinated proteins (18, 46, 47). SIRT1 plays a critical role in maintaining autophagic homeostasis (50). Autophagy is a cellular degradation process involving organelles such as mitochondria and lysosomes, and it significantly contributes to the regulation of exosome generation and secretion (51, 52). Miao et al. (53) reported that cigarette smoke can induce autophagy through reactive oxygen species (ROS)-mediated mechanisms. Recent studies have further demonstrated that cigarette smoke promotes ROS generation, which subsequently disrupts autophagic processes. The regulation of autophagy is closely linked to oxidative stress, which can modulate autophagic activity via both direct and indirect mechanisms (50, 53–55). As an autophagy substrate, SIRT1 undergoes degradation via the autophagosome-lysosome pathway during aging, contributing to autophagy regulation (50, 56). Zhang et al. (57) demonstrated that melatonin protects against sepsis-induced cardiac dysfunction by modulating apoptosis and autophagy through SIRT1 activation. In this study, activation of SIRT1 with SRT1720 was found to upregulate ATG16L1 expression. ATG16L1 is a key autophagy-related protein involved in lysosomal repair and the maintenance of cellular homeostasis. Tan et al. (58) showed that ATG16L1 enhances resistance to cellular toxins by promoting lysosomal exocytosis and membrane protrusion formation, especially in response to membrane damage caused by bacterial pore-forming toxins (PFTs), highlighting its critical role in membrane repair. Autophagy and the endolysosomal system are essential for exosome biogenesis. Impairment of autophagy and lysosomal function can lead to the accumulation of undigested substrates, the formation of aberrant exosomes, and subsequent pathological outcomes (59). The LC3-binding mechanism plays a central role in the autophagy pathway, particularly in the loading and secretion of RNA-binding proteins (RBPs) into extracellular exosomes. This pathway is essential for the regulation of exosomal cargo sorting, particularly for RBPs, and highlights interactions between LC3-binding proteins and other exosomal components, underscoring LC3’s importance in exosome formation and intercellular communication (60, 61). However, dysregulated autophagy can contribute to inflammatory responses. p62 is a central mediator of selective autophagy (62, 63), and its droplet-like structures participate not only in autophagosome formation but also serve as hubs for antioxidant stress responses. The relationship between p62 and autophagy is bidirectional and closely associated with autophagy dysfunction (63, 64). Autophagy encapsulates cytoplasmic material including protein aggregates, damaged organelles, and lipids within double-membrane-enclosed autophagosomes, which subsequently fuse with lysosomes for enzymatic degradation. This lysosome-dependent mechanism supports the renewal of cellular components and preserves cellular integrity. In summary, our findings demonstrate that CSE exposure leads to decreased levels of ATG16L1 and p62, along with elevated LC3B expression, indicating impaired autophagy in treated DCs. These alterations may be linked to changes in the function of DCexos.

In addition to its impact on autophagy, cigarette smoke has been shown to promote necroptosis processes. Here, CSE exposure increased the expression of ZBP1, RIPK3, MLKL, Caspase-8, and Caspase-3 in DCs, coinciding with an imbalance in CD4^+^ T cell differentiation to favor Th17 cells over Tregs in a manner mediated by DCexos. Cigarette smoke can induce DNA damage, and both circulating mitochondrial DNA (cf-mtDNA) and nuclear DNA (cf-nDNA) levels have been reported to be elevated in patients with COPD. Z-DNA is known to trigger ZBP1-dependent necroptosis and inflammation (19, 65–67). Lu et al. (68) showed that cigarette smoke induces necroptosis in COPD. Giordano et al. (65) found increased levels of cell-free mitochondrial DNA in the plasma of COPD patients and in the serum of mice with cigarette smoke-induced emphysema. Chen D et al. (69) further demonstrated that necroptosis-related markers, including RIP3 and phosphorylated MLKL (p-MLKL), are significantly upregulated in lung tissue from both COPD patients and cigarette smoke-exposed mouse models. Cleaved caspase-3 was also sporadically detected in COPD patients. Importantly, inhibition of the RIP3/MLKL pathway attenuated emphysema and reduced inflammatory responses in these models. Wei et al. (70) reported that in cerebral ischemia/reperfusion injury (CIRI), SIRT1 expression was significantly reduced, while RIP1 and other necroptosis-associated proteins were upregulated. Co-immunoprecipitation and immunofluorescence analyses revealed a weakened interaction between SIRT1 and RIP1, promoting necroptotic complex formation and exacerbating cellular necroptosis. These findings suggest that SIRT1 mitigates mitochondrial fragmentation and necroptosis in CIRI via modulation of the RIP1 pathway. The release and function of exosomes are associated not only with autophagy but also with necroptosis. Activated MLKL translocates to the lysosomal membrane and polymerizes to form pores, increasing lysosomal membrane permeability (LMP). This facilitates the rapid release of lysosomal contents, particularly cathepsin B (CTSB), a key mediator of necroptosis and an important factor in vesicle transport and exosome biogenesis. MLKL has been implicated in both the generation and trafficking of exosomes (71, 72). Gupta et al. (73) reported a significant increase in extracellular vesicle release during necrosis in mouse embryonic fibroblasts, with RIPK3 playing a central role. Moreover, extracellular vesicles from necrotic cells contain higher levels of proteins such as RIPK3 and MLKL compared to those from healthy cells. Preyat et al. (74) also found that the NAD+-dependent deacetylase sirtuin family plays a regulatory role in TNF-induced necroptosis. Collectively, these findings suggest that CSE exposure induces necroptosis-associated changes in DC-derived exosome formation and function. Notably, treatment with SRT1720 was shown to reduce necroptosis and modulate the activity of DCs-derived exosomes under CSE exposure, thereby restoring the balance of CD4^+^ T cell differentiation into Th17 and Treg subsets. Whether SIRT1 alleviates necroptosis by regulating autophagy or by reducing mtDNA levels in DCs under CSE exposure will require further investigation.

Autophagy is not only essential for cell survival but also interacts closely with necroptosis (75). These two processes are interconnected and often co-regulated. Following CSE exposure, DCs exhibited elevated expression levels of ZBP1, RIPK3, MLKL, Caspase-8, and Caspase-3, along with increased LC3B expression. In contrast, ATG16L1 and p62 levels were decreased. Reactive oxygen species (ROS) are known to activate both necroptosis and autophagy (76–79). ATG16L1, a component of the ATG12-ATG5/ATG16L1 complex, is critical for the lipidation of LC3, an essential step in autophagosome formation. Moreover, ATG16L1 is involved in intracellular trafficking and the regulation of membrane dynamics, thereby supporting cellular homeostasis. Bai et al. (80) demonstrated that ATG16L1-deficient macrophages display impaired LC3B lipidation and ROS accumulation, which in turn activates the NLRP3 inflammasome and amplifies inflammatory responses. Dysregulated or excessive autophagy may lead to either apoptosis or necroptosis, depending on the cell’s ATP status. When ATP levels drop below 50%, autophagy may instead promote necroptosis (63). Harris et al. (81) found that RIP3 deficiency impairs autophagic flux, resulting in autophagosome accumulation. In inflammatory bowel disease, ATG16L1 has been shown to inhibit RIPK3-dependent necroptosis, thereby activating mitophagy, maintaining mitochondrial integrity, and reducing inflammation (82). Dera et al. (83) further reported that CSE induces autophagy/mitophagy-dependent necroptosis in human bronchial epithelial cells. Another study demonstrated that cigarette smoke can activate both autophagy and necroptosis through the PINK1 pathway in lung epithelial cells (84). Wu et al. (85) observed that MLKL expression and its translocation are closely associated with autophagic flux, revealing a novel co-regulatory relationship between necroptosis and autophagy signaling pathways. Additionally, MLKL, a key necroptotic effector, regulates both the formation and nuclear transport of exosomes and is modulated via RIPK3-mediated phosphorylation (86, 87). Huang et al. (88) identified LC3-interacting region (LIR) motifs within the protein sequences of RIPK1 and RIPK3 in cardiomyocytes. Co-immunoprecipitation assays confirmed direct interactions between LC3 and both RIPK1 and RIPK3. Under hypoxic conditions, LC3 overexpression promotes necroptosis, whereas under normoxic conditions, impaired autophagic flux results in elevated RIPK1 and RIPK3 levels, subsequently triggering necroptosis (89). Mizumura et al. (90) showed that cigarette smoke induces mitophagy-dependent necroptosis in epithelial cells, contributing to lung emphysema pathogenesis. These findings indicate a bidirectional regulatory relationship between autophagy and necroptosis, where each can influence the other. Both mitophagy and general autophagy can be activated through the PINK1/Parkin pathway and are further interconnected via PINK1 and ULK1 signaling. Necroptosis may also influence autophagy: RIPK3 can activate ULK1 and Beclin-1 through AMPK phosphorylation, thereby inducing autophagy, while MLKL directly regulates autophagic processes. Both autophagy and necroptosis intersect with the cGAS-STING pathway (91), although the precise molecular mechanisms underlying these interactions remain to be fully elucidated.

## Conclusions

In summary, our data demonstrate that CSE exposure induces the release of DCexos that polarize CD4^+^ T cells, leading to an imbalance between Th17 and Treg subsets. Treatment with the SIRT1 activator SRT1720 was shown to modulate these exosomes, thereby attenuating the Th17/Treg imbalance. CSE exposure was also found to downregulate SIRT1 expression and promote necroptosis in DCs, accompanied by abnormal autophagy. Importantly, activation of SIRT1 by SRT1720 reduced necroptosis and regulated autophagy in DCs under CSE exposure.

## Data Availability

The original contributions presented in the study are included in the article/supplementary files, further inquiries can be directed to the corresponding author/s.
